# Serum Hepatocyte Growth Factor and Cancer Mortality in an Apparently Healthy Japanese Population

**DOI:** 10.2188/jea.JE20110121

**Published:** 2012-09-05

**Authors:** Maki Otsuka, Hisashi Adachi, David R. Jacobs, Yuji Hirai, Mika Enomoto, Ako Fukami, Shun-ichi Kumagae, Yasuki Nanjo, Kuniko Yoshikawa, Eishi Esaki, Eita Kumagai, Kanako Yokoi, Kinuka Ogata, Eri Tsukagawa, Akiko Kasahara, Kyoko Ohbu, Tsutomu Imaizumi

**Affiliations:** 1 Department of Internal Medicine, Division of Cardio-Vascular Medicine, Kurume University School of Medicine, Kurume, Fukuoka, Japan; 1 久留米大学医学部、内科学講座、心臓・血管内科部門; 2 Department of Community Medicine, Kurume University School of Medicine, Kurume, Fukuoka, Japan; 2 久留米大学医学部、地域医療連携講座; 3 University of Minnesota, School of Public Health, Division of Epidemiology, Minneapolis, Minnesota, USA; 3 ミネソタ大学、公衆衛生学、疫学部門

**Keywords:** cytokine, prospective study, Seven Countries Study, mortality, cancer

## Abstract

**Background:**

In patients with cancer, hepatocyte growth factor (HGF) is elevated and is a predictor of prognosis. We investigated whether serum HGF was a predictive marker for cancer death in a population of community-dwelling Japanese.

**Methods:**

We studied 1492 apparently healthy Japanese adults who underwent health examinations in 1999. Those who reported a history of liver disease or malignancy on a baseline questionnaire were excluded, and plasma HGF was measured in the remaining 1470 participants, who were followed periodically for 10 years. Multivariate proportional hazards regression was used to estimate cancer mortality.

**Results:**

A total of 169 participants died during follow-up (61 from cancer, 32 from cerebrocardiovascular disease, and 76 from other diseases). Mean HGF at baseline was significantly higher among decedents than among survivors (0.26 ± 0.11 vs 0.23 ± 0.09 ng/ml, respectively; *P* < 0.01). The Cox proportional hazards model showed that age, systolic blood pressure, HGF (hazard ratio, 1.27; 95% CI, 1.06–1.52; *P* = 0.009), albumin level, smoking status, and creatinine were independent predictors of all-cause death. Age, HGF (hazard ratio, 1.31; 95% CI, 1.04–1.65; *P* = 0.02), and total cholesterol were independent predictive markers for cancer death.

**Conclusions:**

Serum HGF was a predictor of cancer death in an apparently healthy population of community-dwelling Japanese.

## Abstract (ja)

**<背景>:**

肝細胞増殖因子（Hepatocyte growth factor；HGF）はがん患者においてその血中濃度が上昇し、予後予測因子のひとつである。我々は我が国における大規模な一般住民検診に於いて血清HGF濃度を測定し、将来のがん死亡の予知因子となりうるかどうかを検討した。

**<方法>:**

1999年に行った検診受診者1492名を対象とした。ベースライン時に明らかな肝疾患の既往や最近のがんの既往のあるものを除外した1470名を10年間前向きに追跡した。

**<結果>:**

追跡期間中に169名の死亡が確認された。そのうち61名が癌死亡、32名が心血管死亡、76名がその他の死亡であった。生死別に血清HGF濃度を比較したところ死亡者で有意に高値であった。（死亡者0.26±0.11、生存者0.23±0.09ng/ml,p<0.01）

またCoxの比例ハザードモデルを用いて全死亡との関連性について検討したところ年齢、収縮期血圧、HGF（ハザード比1.27；95%信頼区間1.06-1.52,p=0.009)、アルブミン、喫煙、クレアチニンが独立した全死亡の予測因子であった。さらに年齢、HGF（ハザード比1.31；95%信頼区間1.04-1.65,p=0.02)、総コレステロールは独立したがん死亡の予測因子であった。

**<結論>:**

血清HGF濃度は我が国における健康な一般住民におけるがん死亡の予測因子として有用である可能性を示唆することができた。

## INTRODUCTION

Hepatocyte growth factor (HGF) was discovered in 1984,^1^ purified and isolated in 1986,^2^^,^^3^ and first characterized as a strong mitogen for hepatocytes. It is produced in a number of organs and is now known to be a multifunctional factor with various biological activities.^4^^–^^6^ HGF was found to be elevated in serum from patients with liver disease and cancer, including those with malignancies of the breast,^7^ stomach,^8^^,^^9^ colorectum,^10^ and lung.^11^ Furthermore, HGF was inversely correlated with survival time in cancer patients,^12^ which indicates that it might be a prognostic marker in such patients.^10^^,^^13^ However, existing reports are limited to patients with diagnosed cancer. Thus, we examined whether serum HGF was a predictor of cancer death among apparently healthy Japanese living in the general community.

## METHODS

A periodic epidemiologic survey was performed in 1999 in the small farming community of Tanushimaru, Japan. As reported previously, the demographic characteristics of the residents of this area are similar to those of the general Japanese population.^14^ A total of 1492 adults aged 40 years or older underwent health check-ups, and a questionnaire was used to ascertain their medical history (particularly cancer), use of alcohol, smoking, and current use of medications for hypertension, hyperlipidemia, and diabetes. Alcohol intake and smoking were classified as current habitual use or not. Use of medication for hypertension, hyperlipidemia, and diabetes was coded as dummy variables. Body mass index (BMI) was calculated from measurements of height and body weight. Blood pressure was measured twice with participants in the supine position. A second blood pressure reading was taken after 5 deep breaths, and the fifth-phase diastolic pressure was recorded and used in the analysis. Blood samples obtained from the antecubital vein were centrifuged and frozen. Using these samples, we measured serum glycosylated hemoglobin A1c (Japan Diabetes Society; HbA1c [JDS]), lipids (total cholesterol, high-density lipoprotein [HDL]-cholesterol, and triglycerides), blood urea nitrogen (BUN), creatinine, uric acid, albumin, C-reactive protein (CRP), and liver enzymes (alanine aminotransferase [ALT], aspartate aminotransferase [AST], and γ-glutamyl transpeptidase [γ-GTP]). Plasma HGF was measured by enzyme-linked immunosorbent assay (ELISA).^15^ Intra- and inter-assay coefficients of variation of HGF, as determined by a commercially available laboratory (Kyodo Igaku Laboratory, Fukuoka, Japan), were 1.0% and 3.0%, respectively. The details of HGF measurement have been previously described.^16^ Twenty-four participants who had a history of liver disease, lung disease, or cancer were excluded from the analysis. Ultimately, 1470 participants (595 men and 875 women) were enrolled.

The follow-up period was 10 years. Cause of death was determined on the basis of a review of obituaries, medical records, death certificates, and hospital charts, as well as interviews with primary care physicians, the families of the deceased, and other witnesses. Because many patients with cancer ultimately die from infection or other illnesses, great care was taken to identify underlying cause of death. The information was coded independently according to the rules of the Seven Countries Study^17^ and the World Health Organization’s 10th Revision of the International Statistical Classification of Diseases and Related Health Problems (WHO-ICD).^18^ Follow-up data through the end of March 2010 were analyzed. The follow-up rate was 92.9%.

This study was approved by the Ukiha Branch of the Japan Medical Association, by the local citizens’ committee of Tanushimaru, and by the Ethics Committee of Kurume University. All participants gave informed consent.

### Statistical analysis

Results are presented as mean ± SD. Because of skewed distributions, natural logarithmic transformations were performed for CRP, triglycerides, and γ-GTP. These variables are shown in the original scale in the tables, after analysis using the log (natural)-transformed values. In Tables 1 and 2, the values for CRP, triglycerides, and γ-GTP are presented as geometric mean and range.

**Table 1.** Characteristics of participants by quartile of HGF

Variables	Quartile of HGF level												*P* for trend^a^

	Q1 (≤0.16 ng/ml)			Q2 (0.17–0.21 ng/ml)			Q3 (0.22–0.27 ng/ml)			Q4 (≥0.28 ng/ml)			
			
	No.	%	Mean (SD)	No.	%	Mean (SD)	No.	%	Mean (SD)	No.	%	Mean (SD)	
Total No.	349	24		397	27		341	23		383	26		
No. and % of deaths	25	7		36	9		49	14		59	15		0.001
HGF, ng/ml			0.14 (0.02)			0.19 (0.01)			0.24 (0.02)			0.35 (0.08)	
Age, years			62 (10)			63 (11)			63 (11)			63 (11)	0.397
Male sex	93	27		154	39		155	45		193	50		<0.0001
Body mass index^b^			22 (3)			23 (3)			23 (3)			24 (3)	<0.0001
Systolic BP, mm Hg			131 (21)			132 (20)			136 (21)			136 (22)	0.001
Diastolic BP, mm Hg			78 (11)			78 (11)			80 (12)			80 (12)	0.034
HbA1c, %			5.2 (0.7)			5.2 (0.7)			5.3 (0.7)			5.3 (0.9)	0.103
Blood urea nitrogen, mmol/l			5.9 (1.5)			5.7 (1.4)			5.9 (1.5)			5.8 (1.5)	0.323
Creatinine, µmol/l			73 (15)			74 (14)			77 (19)			78 (17)	<0.0001
Uric acid, µmol/l			268 (71)			292 (83)			303 (89)			303 (83)	<0.0001
C-reactive protein^c^, mg/dl			0.17			0.18			0.19			0.23	<0.0001
Range			0.1–3.9			0.1–3.9			0.1–3.9			0.1–11.5	
Albumin, g/l			44 (2)			44 (2)			44 (3)			44 (3)	0.546
Total cholesterol, mmol/l			5.3 (0.9)			5.2 (0.9)			5.3 (1.0)			5.0 (0.9)	<0.001
HDL cholesterol, mmol/l			1.5 (0.4)			1.5 (0.3)			1.5 (0.4)			1.4 (0.4)	<0.0001
Triglycerides^c^, mmol/l			0.99			1.08			1.13			1.20	<0.0001
Range			0.33–7.29			0.33–10.9			0.41–13.5			0.32–14.5	
AST, units/l			27 (4)			28 (4)			28 (6)			29 (6)	0.010
ALT, units/l			26 (3)			26 (3)			26 (3)			27 (4)	0.049
γ-GTP^c^, units/l			15			18			20			23	<0.0001
Range			1–201			1–218			3–896			3–305	
Smoking	30	9		52	13		69	20		87	23		<0.0001
Alcohol	61	17		90	23		74	22		85	22		0.297
Antihypertensive medication	51	15		72	18		71	21		89	23		0.022
Antihyperlipidemic medication	18	5		21	5		19	6		14	4		0.619
Antidiabetic medication	6	2		11	3		8	2		15	4		0.311

Data are mean (SD), geometric mean, range, or percent.

Abbreviations: HGF, hepatocyte growth factor; SD, standard deviation; BP, blood pressure; HbA1c, glycosylated hemoglobin A1c; HDL, high-density lipoprotein; AST, aspartate aminotransferase; ALT, alanine aminotransferase; γ-GTP, γ-glutamyl transpeptidase.

^a^Calculated from linear regression analysis for continuous variables and from logistic regression analysis for dichotomous variables.

^b^Weight (kg)/height (m)^2^.

^c^These variables are shown in the original scale after analysis using log (natural)-transformed values.

**Table 2.** Characteristics of participants stratified by vital status

Variables	Survivors			Decedents			*P* value
	
	No.	%	Mean (SD)	No.	%	Mean (SD)	
Total No.	1291	88		169	12		
HGF, ng/ml			0.23 (0.09)			0.26 (0.11)	0.009
Age, years			61 (10)			72 (9)	<0.0001
Male sex	487	38		103	61		<0.0001
Body mass index^a^			23 (3)			22 (3)	0.016
Systolic BP, mm Hg			132 (20)			144 (24)	<0.0001
Diastolic BP, mm Hg			79 (11)			81 (12)	0.056
HbA1c, %			5.2 (0.7)			5.4 (1.2)	0.033
Blood urea nitrogen, mmol/l			5.7 (1.4)			6.4 (1.9)	<0.0001
Creatinine, µmol/l			74 (15)			85 (22)	<0.0001
Uric acid, µmol/l			286 (83)			321 (89)	<0.0001
C-reactive protein^b^, mg/dl			0.19			0.22	0.0004
Range			0.1–11.5			0.1–5.2	
Albumin, g/l			44 (2)			42 (3)	<0.0001
Total cholesterol, mmol/l			5.2 (0.9)			4.9 (0.9)	<0.0001
HDL cholesterol, mmol/l			1.5 (0.4)			1.5 (0.4)	0.485
Triglycerides^b^, mmol/l			1.10			1.09	0.837
Range			0.32–14.4			0.34–4.73	
AST, units/l			28 (5)			28 (6)	0.485
ALT, units/l			26 (4)			26 (3)	0.460
γ-GTP^b^, units/l			18			22	0.043
Range			1–310			3–896	
Smoking	187	15		47	28		<0.0001
Alcohol	268	21		40	24		0.429
Antihypertensive medication	234	18		48	28		0.02
Antihyperlipidemic medication	60	5		12	7		0.228
Antidiabetic medication	32	2		8	5		0.148

Data are mean (SD), geometric mean, range, or percent.

Abbreviations: HGF, hepatocyte growth factor; SD, standard deviation; BP, blood pressure; HbA1c, glycosylated hemoglobin A1c; HDL, high density lipoprotein; AST, Aspartate aminotransferase; ALT, Alanine aminotransferase; γ-GTP, γ-glutamyl transpeptidase.

^a^Weight (kg)/height (m)^2^.

^b^These variables are shown in the original scale after analysis using log (natural)-transformed values.

Mean HGF level was classified into quartiles as follows: ≤0.16 ng/ml, 0.17–0.21 ng/ml, 0.22–0.27 ng/ml, and ≥0.28 ng/ml. Analysis of variance was used to compare the means of variables, stratified by quartile of HGF levels. Differences between the 2 groups (survivors vs decedents) were assessed using the *t* test. The χ^2^ test was used to test differences between groups in categorical variables.

Multivariate proportional hazards regression was used to estimate the predictive HGF level for all-cause death and cancer death. We estimated hazard ratios (HRs) and their 95% CIs per 1-unit (approximately 1 SD) increase in the variable. Survival curves for all-cause death and cancer death for each HGF quartile were estimated with the Kaplan-Meier method and interpreted using the log-rank statistic. Statistical significance was defined as a *P* value less than 0.05. All statistical analyses were performed using SAS software (Release 9.2, SAS Institute, Cary, NC, USA).

## RESULTS

### Baseline characteristics associated with HGF

Participants were divided into quartiles according to HGF level. There was a significant positive association between HGF and mortality. In addition, HGF was significantly positively associated with male sex, BMI, systolic and diastolic blood pressures, creatinine, uric acid, CRP, triglycerides, AST, ALT, γ-GTP, smoking, and use of antihypertensive medication and significantly inversely associated with total cholesterol and HDL cholesterol (Table 1).

### Cause of death and characteristics of decedents

We were able to ascertain cause of death for 92.9% of deaths. There were 169 (103 men and 66 women) deaths: 61 (36.1%) from cancer, 32 (18.9%) from cerebrocardiovascular disease, 27 (16.0%) from infection, and 49 (29.0%) from other causes.

Table 2 shows the characteristics of participants stratified by vital status. Factors positively associated with death were age, male sex, systolic blood pressure, HbA1c, BUN, creatinine, uric acid, CRP, γ-GTP, smoking, use of antihypertensive medication, and HGF, whereas BMI, albumin, and total cholesterol were inversely associated with death.

**Figure 1.**
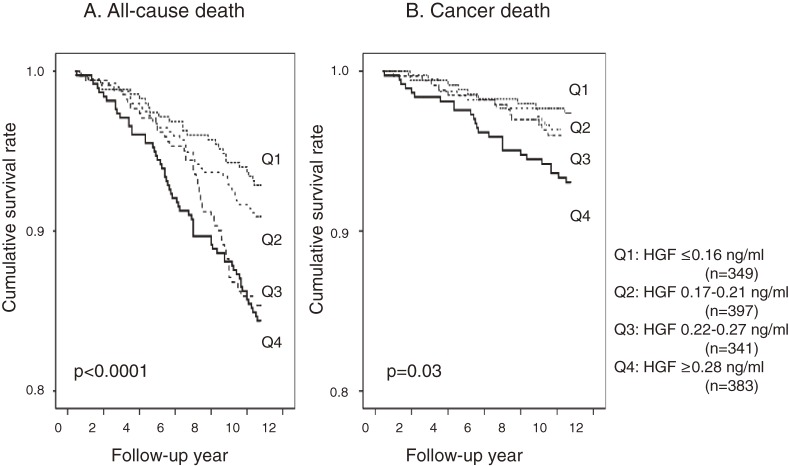
(A) Kaplan-Meier survival curves for all-cause death, stratified by quartile of hepatocyte growth factor (HGF), after adjustment for age and sex. (B) Kaplan-Meier survival curves for cancer death, stratified by quartile of hepatocyte growth factor (HGF), after adjustment for age and sex.

Regression coefficients for all-cause death in the univariate proportional hazards regression model are shown in Table 3. Age, male sex, systolic blood pressure, HbA1c, BUN, creatinine, uric acid, CRP, γ-GTP, smoking, antihypertensive medication, and HGF were significant positive predictors of all-cause death, whereas BMI, albumin, and total cholesterol were inversely associated with all-cause death. Figure 1A shows the cumulative survival curves for all-cause death stratified by HGF quartile in univariate analysis. The HRs by HGF quartile were 1.30 (95% CI, 0.77–2.22) for Q2, 2.18 (1.31–3.62) for Q3, and 2.41 (1.47–3.94) for Q4. Cancer death was positively associated with age, male sex, systolic blood pressure, HbA1c, BUN, creatinine, and smoking and inversely associated with albumin and total cholesterol (Table 4). Figure 1B shows the cumulative survival curves for cancer death stratified by HGF quartile in univariate analysis. Mortality increased in relation to HGF (log-rank test = 11.3; *P* < 0.001). The HRs by HGF quartile were 1.39 (95% CI, 0.60–3.26) for Q2, 1.39 (0.58–3.35) for Q3, and 2.72 (1.25–5.92) for Q4. For cancer death, mortality increased in relation to HGF (log rank test = 8.23; *P* = 0.03).

**Table 3.** Univariate regression coefficients for all-cause death from a Cox proportional hazards regression model

Variable (increment)	Beta	SE	Hazard ratio	95% CI	*P* value
HGF (0.09 ng/ml)	3.271	0.721	1.35	1.19, 1.54	<0.0001
Age (10 years)	0.103	0.008	2.95	2.49, 3.49	<0.0001
Male sex	0.835	0.185	2.31	1.60, 3.32	<0.0001
Body mass index^a^ (3 kg/m^2^)	−0.067	0.025	0.81	0.69, 0.95	0.009
Systolic BP (20 mm Hg)	0.021	0.003	1.57	1.38, 1.79	<0.0001
Diastolic BP (11 mm Hg)	0.012	0.006	1.15	0.99, 1.34	0.060
HbA1c (0.7%)	0.210	0.069	1.18	1.06, 1.31	0.002
Blood urea nitrogen (1.4 mmol/l)	0.089	0.016	1.44	1.26, 1.65	<0.0001
Creatinine (16.4 µmol/l)	1.452	0.168	1.30	1.22, 1.38	<0.0001
Uric acid (59.4 µmol/l)	0.244	0.050	1.40	1.22, 1.61	<0.0001
C-reactive protein^b^ (1.8 times)	0.328	0.104	1.39	1.13, 1.70	0.002
Albumin (2 g/l)	−2.253	0.268	0.56	0.49, 0.64	<0.0001
Total cholesterol (0.87 mmol/l)	−0.011	0.002	0.68	0.58, 0.80	<0.0001
HDL cholesterol (0.36 mmol/l)	−0.003	0.005	0.99	0.82, 1.11	0.557
Triglycerides^b^ (1.1 times)	−0.038	0.153	0.96	0.71, 1.30	0.804
AST (5 units/l)	0.011	0.014	1.06	0.91, 1.24	0.447
ALT (3 units/l)	−0.020	0.027	0.93	0.77, 1.13	0.463
γ-GTP^b^ (2.2 times)	0.224	0.102	1.25	1.03, 1.53	0.030
Smoking	0.785	0.172	2.19	1.57, 3.07	<0.0001
Alcohol	0.172	0.181	1.19	0.83, 1.69	0.341
Antihypertensive medication	0.545	0.170	1.73	1.23, 2.41	0.001
Antihyperlipidemic medication	0.428	0.299	1.54	0.85, 2.76	0.152
Antidiabetic medication	0.592	0.362	1.81	0.89, 3.68	0.102

Abbreviations: HGF, hepatocyte growth factor; BP, blood pressure; HDL, high-density lipoprotein; AST, aspartate aminotransferase; ALT, alanine aminotransferase; γ-GTP, γ-glutamyl transpeptidase.

^a^Weight (kg)/height (m)^2^.

^b^These variables are shown in the original scale after analysis using log (natural)-transformed values. These values indicate hazard ratios per times increase.

**Table 4.** Univariate regression coefficients for cancer death from a Cox proportional hazards regression model

Dependent variable (increment)	Beta	SE	Hazard ratio^c^	95% CI	*P* value
HGF (0.09 ng/ml)	3.949	1.185	1.44	1.16, 1.78	<0.001
Age (10 years)	0.072	0.013	2.14	1.62, 2.82	<0.0001
Male sex	0.919	0.295	2.51	1.41, 4.47	0.001
Body mass index^a^ (3 kg/m^2^)	−0.005	0.042	0.98	0.76, 1.27	0.894
Systolic BP (20 mm Hg)	0.013	0.005	1.31	1.04, 1.66	0.024
Diastolic BP (11 mm Hg)	0.009	0.011	1.11	0.87, 1.43	0.413
HbA1c (0.7%)	0.280	0.100	1.24	1.07, 1.45	0.005
Blood urea nitrogen (1.4 mmol/l)	0.058	0.029	1.27	1.01, 1.61	0.044
Creatinine (16.4 µmol/l)	1.042	0.409	2.84	1.27, 6.32	0.011
Uric acid (59.4 µmol/l)	0.101	0.090	1.15	0.90, 1.47	0.259
C-reactive protein^b^ (1.8 times)	0.320	0.174	1.38	0.98, 1.94	0.066
Albumin (2 g/l)	−1.828	0.471	0.63	0.49, 0.79	<0.001
Total cholesterol (0.87 mmol/l)	−0.015	0.004	0.58	0.44, 0.76	<0.0001
HDL cholesterol (0.36 mmol/l)	−0.009	0.009	0.87	0.67, 1.14	0.327
Triglycerides^b^ (1.1 times)	−0.019	0.257	0.98	0.59, 1.62	0.940
AST (5 units/l)	−0.007	0.031	0.96	0.69, 1.33	0.813
ALT (3 units/l)	−0.033	0.051	0.89	0.63, 1.26	0.508
γ-GTP^b^ (2.2 times)	0.123	0.181	1.13	0.79, 1.61	0.496
Smoking	0.828	0.288	2.29	1.30, 4.03	0.004
Alcohol	0.422	0.287	1.53	0.87, 2.68	0.141
Antihypertensive medication	−0.127	0.347	0.88	0.45, 1.74	0.714
Antihyperlipidemic medication	0.621	0.467	1.86	0.75, 4.66	0.183
Antidiabetic medication	0.951	0.517	2.59	0.94, 7.15	0.066

Abbreviations: SE, standard error; HGF, hepatocyte growth factor; BP, blood pressure, HDL, high-density lipoprotein, AST, aspartate aminotransferase; ALT, alanine aminotransferase; γ-GTP, γ-glutamyl transpeptidase.

^a^Weight (kg)/height (m)^2^.

^b^These variables are shown in the original scale after analysis using log (natural)-transformed values. These values indicate hazard ratios per times increase.

^c^Hazard ratio per 1-increment increase in variable.

Using the significant factors shown in Tables 3 and 4, we performed multivariate proportional hazards regression analysis (Tables 5 and 6). Age, systolic blood pressure, HGF, albumin (inversely), smoking, and creatinine remained significantly associated with all-cause death (Table 5): the HRs by HGF quartile after adjustment for confounding factors were 1.15 (95% CI, 0.68–1.93) for Q2, 1.49 (0.91–2.44) for Q3, and 1.77 (1.10–2.97) for Q4. For cancer death, age, HGF, and total cholesterol (inversely) remained significant (Table 6): after adjustment for confounding factors, the HRs by HGF quartile were 1.22 (95% CI, 0.53–2.83) for Q2, 1.19 (0.50–2.83) for Q3, and 2.21 (1.03–4.76) for Q4.

**Table 5.** Multivariate proportional hazards regression analysis of all-cause death

Dependent variable (increment)	Beta	SE	Hazard ratio^c^	95% CI	*P* value
Age (10 years)	0.088	0.013	2.53	1.90, 3.37	<0.0001
Systolic BP (20 mm Hg)	0.013	0.005	1.32	1.08, 1.63	0.007
HGF (0.09 ng/ml)	2.593	1.006	1.27	1.06, 1.52	0.009
Albumin (2 g/l)	−1.019	0.460	0.77	0.61, 0.97	0.026
Smoking	0.654	0.298	1.93	1.07, 3.45	0.028
Creatinine (16.4 µmol/l)	0.706	0.311	2.03	1.10, 5.73	0.033
γ-GTP^b^ (2.2 times)	0.072	0.110	1.19	0.96, 1.48	0.109
C-reactive protein^b^ (1.8 times)	0.160	0.106	1.17	0.96, 1.44	0.128
Hypertensive medication	−0.382	0.263	0.68	0.41, 1.14	0.146
Blood urea nitrogen (1.4 mmol/l)	0.038	0.027	1.17	0.94, 1.46	0.161
HbA1c (0.7%)	0.133	0.114	1.11	0.93, 1.32	0.242
Total cholesterol (0.87 mmol/l)	−0.002	0.003	0.91	0.71, 1.17	0.455
Uric acid (59.4 µmol/l)	0.040	0.086	1.06	0.84, 1.34	0.639
Male sex	0.107	0.296	1.11	0.62, 1.99	0.716
Body mass index^a^ (3 kg/m^2^)	0.003	0.038	1.01	0.80, 1.28	0.924

Abbreviations: HGF, hepatocyte growth factor; SE, standard error; BP, blood pressure; γ-GTP, γ-glutamyl transpeptidase.

^a^Weight (kg)/height (m)^2^.

^b^These variables are shown in the original scale after analysis using log (natural)-transformed values. These values indicate hazard ratios per times increase.

^c^Hazard ratio per 1-increment increase in variable.

**Table 6.** Multivariate proportional hazards regression analysis of cancer death

Dependent variable (increment)	Beta	SE	Hazard ratio^a^	95% CI	*P* value
Age (10 years)	0.065	0.017	2.00	1.39, 2.87	<0.001
HGF (0.09 ng/ml)	2.929	1.267	1.31	1.04, 1.65	0.020
Total cholesterol (0.87 mmol/l)	−0.010	0.005	0.68	0.48, 0.97	0.034
Blood urea nitrogen (1.4 mmol/l)	0.059	0.034	1.28	0.97, 1.68	0.082
HbA1c (0.7%)	0.193	0.123	1.16	0.96, 1.41	0.116
Male sex	0.547	0.408	1.73	0.78, 3.85	0.180
Albumin (2 g/l)	−0.787	0.630	0.82	0.59, 1.12	0.211
Smoking	0.472	0.383	1.60	0.76, 3.40	0.217
Creatinine (16.4 µmol/l)	0.506	0.567	1.66	0.55, 5.04	0.371
Systolic BP (20 mm Hg)	0.003	0.007	1.08	0.81, 1.45	0.608

Abbreviations: HGF, hepatocyte growth factor; SE, standard error; BP, blood pressure.

^a^Hazard ratio per 1-increment increase in variable.

There was no significant association between HGF and cerebrocardiovascular death (data not shown).

## DISCUSSION

We investigated whether plasma HGF predicted cancer death among a population of Japanese adults. Our results showed that plasma HGF level was predictive of both all-cause death and cancer death.

We made every effort in this prospective study to ascertain cause of death, and the death rates among this population were comparable to those of the Japanese general population.^19^ Multivariate proportional hazards regression analysis revealed that age, systolic blood pressure, HGF, albumin (inversely), smoking, and creatinine were independently associated with all-cause death (Table 5). Age, blood pressure, and smoking are known causes of cerebrocardiovascular death. Thus, it is feasible that these factors contributed to all-cause death in this population. However, the number of deaths from cerebrocardiovascular disease was too small to analyze, and we were thus unable to identify the factors responsible for cerebrocardiovascular death in this study. In addition to the above-mentioned factors, low albumin and HGF were significant predictors of all-cause death (Table 5). Low albumin may indicate poor nutritional status. The predictive power of HGF was comparable to that of systolic blood pressure (Table 5). At present, it is unclear why HGF was a significant predictor of all-cause death.

We investigated whether HGF was a predictor of cancer death in this apparently healthy population because cancer is the most common cause of death in this population and because HGF level was found to be a predictor of death in patients with cancer.^8^^–^^13^ Multivariate proportional hazards regression analysis revealed that age, HGF, and cholesterol (inversely) were predictors of cancer death. Although baseline HGF was associated with cholesterol (inversely) and some other factors, as shown in Table 1, the data in Table 6 show that HGF was a significant independent factor for cancer death, which indicates that HGF is a predictor for cancer death in apparently healthy community-dwelling Japanese adults.

The pathophysiologic mechanisms for the association between HGF and cancer death in this population were not investigated in this epidemiologic study. Because we excluded patients with history of cancer at baseline, it is natural to assume that the remaining study participants developed cancer during follow-up. To confirm this, we divided cancer deaths into those that occurred during the first 5 years of follow-up and those that occurred in the second 5 years of follow-up. There were 30 cancer deaths during the first 5 years and 31 during the second 5 years. As mentioned above, serum HGF is elevated in individuals with cancer.^7^^–^^11^ HGF is released from cancer cells^11^^,^^12^ and stimulates not only tumor cell invasion but also neovascularization as a potent endothelial growth factor.^13^^,^^20^^–^^23^ If participants who died within 5 years had subclinical cancer at baseline, they might have had higher baseline HGF levels. However, we found that baseline HGF was similar in the early and late study periods among participants who died of cancer. Thus, the increase in the risk of cancer death associated with high HGF was not due to the presence of subclinical cancer. It is possible that participants with high baseline HGF levels experienced rapid growth and spread of cancer once they developed cancer. However, this hypothesis cannot be confirmed in an observational study.

A strength of the present study is that it is the first prospective cohort study to show that HGF is a predictor of cancer death in apparently healthy adults. Moreover, the follow-up rate was high (92.9%).

A limitation of this study is that, because of the relatively small number of cancer deaths, we were unable to investigate the association between HGF and particular malignancies. Second, the cancer endpoint was not incidence but mortality. Third, the association between HGF and cancer death might be confounded by several factors. However, this association remained significant after adjustment for confounders.

In conclusion, serum HGF was a predictor of cancer death in an apparently healthy community-dwelling Japanese population. Our findings suggest that serum HGF may be a predictive marker for cancer death in the general population.

## ONLINE ONLY MATERIALS

Abstract in Japanese.
