# Supplementary material for: Serum Hepatocyte Growth Factor and Cancer Mortality in an Apparently Healthy Japanese Population
Source: J Epidemiol. 2012 Sep 5;22(5):395–401. doi: 10.2188/jea.JE20110121 (PMC3798633; doi:10.2188/jea.JE20110121)
Supplement: Abstract in Japanese. [file je-22-395-s001.pdf]

一般住民における血清 HGF 濃度とがん死亡との関連性の検討

大塚麻樹[1]、足達　寿[1,2]、 David R. Jacobs, Jr[3]、 平井祐治[1]、 榎本美佳[1]、深水亜子[1]、
熊谷俊一[1]、南條泰輝[1]、吉川邦子[1]、江崎英司[1]、熊谷英太[1]、横井加奈子[1]、緒方絹歌[1]、
塚川絵理[1]、笠原明子[1]、大部恭子[1]、今泉　勉[1]

[1] Department of Internal Medicine, Division of Cardio-Vascular Medicine, Kurume University School of Medicine （久留米大学医学部、内科学講座、心臓・血管内科部門）
[2] Department of Community Medicine, Kurume University School of Medicine
（久留米大学医学部、地域医療連携講座）
[3] Division of Epidemiology, School of Public Health, University of Minnesota
（ミネソタ大学、公衆衛生学、疫学部門）

〈背景〉肝細胞増殖因子（Hepatocyte growth factor ; HGF）はがん患者においてその血中濃度が上昇し、予後予測因子のひとつである。我々は我が国における大規模な一般住民検診に於いて血清 HGF 濃度を測定し、将来のがん死亡の予知因子となりうるかどうかを検討した。

〈方法〉1999 年に行った検診受診者 1492 名を対象とした。ベースライン時に明らかな肝疾患の既往や最近のがんの既往のあるものを除外した 1470 名を 10 年間前向きに追跡した。

〈結果〉追跡期間中に 169 名の死亡が確認された。そのうち 61 名が癌死亡、32 名が心血管死亡、76 名がその他の死亡であった。生死別に血清 HGF 濃度を比較したところ死亡者で有意に高値であった。（死亡者 0.26±0.11、生存者 0.23±0.09ng/ml, $p<0.01$）
また Cox の比例ハザードモデルを用いて全死亡との関連性について検討したところ年齢、収縮期血圧、HGF（ハザード比 1.27 ; 95%信頼区間 1.06-1.52, p=0.009）、アルブミン、喫煙、クレアチニンが独立した全死亡の予測因子であった。さらに年齢、HGF（ハザード比 1.31 ; 95%信頼区間 1.04-1.65, p=0.02）、総コレステロールは独立したがん死亡の予測因子であった。

〈結論〉血清 HGF 濃度は我が国における健康な一般住民におけるがん死亡の予測因子として有用である可能性を示唆することができた。

キーワード : サイトカイン、前向き調査、世界 7 か国共同研究、死亡率、がん
